# Structured digital health intervention for leprosy awareness: a case study of analyzing public engagement and sentiment in a Sri Lankan leprosy awareness campaign on Facebook

**DOI:** 10.1186/s12879-026-13355-x

**Published:** 2026-04-18

**Authors:** Millawage Supun Dilara Wijesinghe, Ashan Pathirana, Upeksha Gayani Karawita, Nissanka Achchi Kankanamalage Ayoma Iroshanee Nissanka, Aruni Indika Samarathunga Hewapathirana, Indira Padmapani Kahawita, K. D. N. Prasad Ranaweera, A. M. M. A. P. Alagiyawanna

**Affiliations:** 1 Health Promotion Bureau, Colombo, Sri Lanka; 2 Central Leprosy Clinic, National Hospital of Sri Lanka, Colombo, Sri Lanka; 3 Anti-Leprosy Campaign, Colombo, Sri Lanka; 4 No. 02, Kynsey Road, Colombo, 10 Sri Lanka

**Keywords:** Leprosy, Public engagement, Facebook campaign, Stigma, Digital health, Neglected tropical diseases

## Abstract

**Background:**

Leprosy is a curable yet stigmatized disease that persists in Sri Lanka, despite the country having met the World Health Organization (WHO) elimination threshold in 1995. Disruptions in outreach during the COVID-19 pandemic heightened concerns regarding unreported cases, emphasizing the necessity for innovative awareness strategies. Although social media is progressively utilized for health promotion in low- and middle-income nations, there is no understanding of how expert-moderated digital discussions can influence public attitudes and stimulate clinical visits in these countries.

**Methods:**

We employed a single embedded case study of a government-led Facebook awareness post on leprosy, analyzing 806 public comments and replies. Data were examined using reflexive thematic analysis and the framework method to characterize user archetypes, stigma expressions, sentiment shifts, and referral actions. Intervention episodes, where identified medical experts engaged, were compared with non-intervention episodes to assess changes in misinformation, reassurance, and intent to seek care. Language equity was considered by analyzing contributions in Sinhala, Tamil, and English. To enhance trustworthiness, intercoder reliability, analyst triangulation, and ethical safeguards for Internet-mediated research were applied.

**Results:**

In this case, the thread showed features we describe as a ‘digital frontline,’ a public, expert-moderated space that provided triage-like guidance, corrected misinformation, and signposted users to care. The WhatsApp line was followed by more than 150 inbound WhatsApp contacts within 24 h, as reported to the study team by the Anti-Leprosy Campaign, Ministry of Health; however, the downstream clinical outcomes were not independently verified. Expert communication was followed by observable shifts in sentiment from anxiety and confusion to reassurance and gratitude, whereas community amplification appeared to reinforce credibility and limit misinformation. Stigma emerged in two forms: fear-based concerns about contagion and deformity, and normalized humor that trivialized illness and may risked deterring help seeking. A key limitation was the reliance on Sinhala, with limited Tamil engagement despite the high prevalence in Tamil-speaking regions.

**Conclusion:**

This case suggests that expert-moderated social media threads may, in some contexts, take on ‘digital frontline’ features as defined here and may support referral-oriented engagement. Future studies should test whether similar arrangements produce reliable referrals and equitable access across languages when linked to national leprosy services and WHO strategy priorities.

## Introduction

Leprosy remains a curable but stigmatized neglected tropical disease that primarily affects the skin and peripheral nerves of the host. Without timely multidrug therapy (MDT), it can cause preventable disabilities. Current WHO guidance emphasizes both curability and the importance of early detection, while highlighting the ongoing social stigma and discrimination that impede care-seeking [1].

Sri Lanka achieved the WHO elimination threshold (< 1/10,000 prevalence) in 1995; however, transmission persists. A continuing annual burden of roughly 1,600-2,000 new detections in recent years has been reported, necessitating renewed efforts to reach “zero leprosy” [2]. In line with the WHO’s Global Leprosy Strategy 2021–2030, national targets prioritize reducing incidence and Grade-2 disability through integrated case detection, surveillance, and stigma reduction [3, 4].

The COVID-19 pandemic disrupted routine outreach (e.g., mobile skin clinics and active case-finding) across many settings, and Sri Lanka was no exception, prompting concern that undiagnosed disease accumulated and delayed the trajectory toward national goals. The WHO Sri Lanka emphasized accelerating integrated case detection and awareness in the post-pandemic period, and reporting of detection during 2020–2021 with a rebound of > 1,300 new cases in 2022, including pediatric cases [5, 6].

Against this backdrop, digital channels have become an important complement to traditional outreach methods. Contemporary evidence shows that social media campaigns can move knowledge, attitudes, and even behavior when they generate meaningful engagement, reframing the classic hierarchy-of-effects for health communication to put “engagement” at the center. Systematic reviews also document the growing use of social platforms for public health communication and behavior change, including in low- and middle-income countries (LMICs) [7]. Moreover, lightweight messaging tools such as WhatsApp have been deployed as triage/referral conduits in LMIC clinical pathways, demonstrating their acceptability and feasibility for rapid, low-barrier navigation from community to clinic [8].

However, there are still some important evidence gaps. First, while social media are widely used for health messaging, there is limited empirical work in LMICs that traces a full digital-to-clinical pathway in real time, that is, showing how expert-moderated threads convert public engagement into actual clinical consultations for a neglected tropical disease (NTD) such as leprosy [9]. Second, although stigma is well documented in Sri Lanka [10], including emerging local instruments and recent analyses, few studies have characterized how “normalized” trivialization and humor within social threads may suppress help-seeking for early skin signs [11]. Third, multilingual equity is under-addressed despite the Anti Leprosy Campaign (ALC) communication strategy’s identification of awareness gaps and the need for stronger media use, an issue with particular salience for Tamil-speaking high-prevalence areas, as noted in national planning [12].

This study addresses these gaps by analyzing public engagement with a Sri Lankan Ministry of Health Facebook campaign, focusing on how real-time participation by identified medical experts shapes information flow, sentiment, and referrals to a dedicated WhatsApp line. In this paper, we use the term ‘digital frontline’ as an analytic label for what emerged in this thread, a public, expert-moderated discussion that provided triage-like guidance, corrected misinformation, and signposted people to care. We hypothesized that expert participation within a public thread (a) shifts sentiment from anxiety or uncertainty to reassurance, (b) reduces misinformation, and (c) is associated with concrete help-seeking via a low-barrier messaging referral. The objectives were to characterize user archetypes and informational needs, quantify and qualitatively assess the impact of expert responses on sentiment and discourse, evaluate indications of referral-oriented engagement through the WhatsApp pathway, and surface equity considerations, including stigma expression and language accessibility, to inform Sri Lanka’s leprosy communication strategy in alignment with the WHO’s 2021–2030 framework [13, 14].

## Methods

This study employed a retrospective embedded single-case study design to examine how a government-led Facebook awareness thread functions as a real-time public health intervention. The case study methodology was selected to allow an in-depth exploration of a contemporary phenomenon in its natural context, where the boundaries between the intervention (expert-moderated communication) and context (public discourse on a live social platform) are blurred, consistent with established guidance on case study designs. The case comprised one Health Promotion Bureau (Sri Lanka) Facebook post on leprosy and its ensuing public discussion; the embedded units of analysis were individual comment-reply episodes and expert-public interaction sequences [15].

The setting and data source were a public Facebook thread explicitly designed by the national leprosy authorities at the Central Leprosy Clinic and Health Promotion Bureau as a digital health intervention that combined informational messaging with a low-barrier WhatsApp referral pathway. The index post was published on March 22, 2023, on the Health Promotion Bureau Facebook page, and the thread was captured on 26th August 2025 at 10.30 am. Sri Lankan time. The capture cutoff was defined a priori to limit ongoing edits and additions. The interval between the original post (March 2023) and the data capture point allowed observation of the mature trajectory of the discussion. However, as with all retrospective analyses of social media content, the dataset reflects only comments publicly visible at the time of capture. Platform algorithms may influence the visibility and ordering of comments, and some comments may have been edited or deleted prior to capture. Accordingly, the dataset represents a snapshot of the accessible discussion rather than a complete historical record of all interactions. We captured all publicly visible comments and replies available at the cutoff by the online export tool Export Comments™, and stored the dataset spreadsheet after removing the usernames and direct identifiers. For each comment or reply, we recorded the posting timestamp if visible, comment or reply status, language (Sinhala, Tamil, English), whether the account was an individual or an official page, and whether the post contained a question, advice, stigma-related content, or referral intent. Private messages and WhatsApp message content were not accessed for this study. The final corpus comprised 806 comments and replies. This post was selected as a theoretically informative case because it generated substantial public engagement and sustained participation from identified medical experts within the national leprosy program, including dermatology leadership and the Central Leprosy Clinic account. The level of interaction enabled detailed examination of information exchange, sentiment shifts, and referral-oriented responses within the discussion. Consistent with case study methodology, the objective was not statistical generalization but analytic insight into how expert-moderated social media threads may function as a digital engagement and triage space in a public health campaign context. Anti-Leprosy Campaign program monitoring indicated over 150 inbound WhatsApp contacts within the first day, as reported by Dr. K. D. N. Prasad Ranaweera (personal communication, March 2023); we did not access WhatsApp message content or link contacts to clinic records.

The inclusion criteria were all on-thread public comments and replies posted by any account (citizens, official clinic page, named experts) visible at capture; private messages and WhatsApp exchanges were not accessed. We excluded clear spam/unrelated content after independent screening, while retaining light social banter given its analytical relevance to stigma and engagement. Spam or unrelated content was defined as comments that were clearly promotional, automated, duplicated, or unrelated to the topic of leprosy, skin conditions, or the ongoing discussion in the thread. The operationalization of “thread,” “comment,” and “reply” followed the platform’s native structure; interaction episodes were defined as a focal comment and all direct replies within that subthread.

Because the discourse was predominantly in Sinhala with later Tamil contributions and occasional English, all Sinhala/Tamil segments used in the analysis were translated into English by bilingual analysts, with a second researcher cross-checking translations for fidelity of meaning and tone. Where specific vernacular (e.g., colloquialisms for common dermatoses) shaped interpretation, the original term was retained in the analytic memos alongside an agreed gloss. Language patterns were treated as data, given their equity implications for access to care.

Qualitative analysis was conducted in two complementary streams. First, we conducted a reflexive thematic analysis to surface patterned meanings across the corpus, following established steps of familiarization, inductive code generation, theme development, review, definition/naming, and analytic write-up. Second, we applied the Framework Method to matrix the data by role archetype (e.g., expert, anxious inquirer, grateful beneficiary, casual interlocutor), information needs, stigma expressions, and referral actions, enabling systematic within- and across-episode comparisons. The initial codebook blended inductive codes with sensitizing concepts from digital health communication (e.g., engagement, reassurance) and stigma literature; it was iteratively refined during team coding meetings [16].

Given the study aim to understand whether expert participation shaped the discourse, we undertook focused episode analysis of “intervention episodes” in which an identified expert replied within a subthread. A non-intervention episode was defined as a comparable comment reply sequence in which no expert response was present. For each episode, we qualitatively assessed pre- and post-intervention shifts in expressed sentiment (e.g., anxious → reassured), presence/absence of misinformation, and evidence of action orientation (e.g., intent to use clinic/WhatsApp), treating the expert post as the index event. These episodes were contrasted with the matched non-intervention episodes. In total, 31 intervention episodes and 775 non-intervention episodes were examined. The observed patterns (e.g., fear de-escalation, community amplification of expert guidance) were traced back to exemplar episodes for a thick description.

To enhance trustworthiness, we combined analyst triangulation, an auditable analytic trail (versioned codebook, decision logs, and memos), and structured team debriefs. We assessed intercoder reliability on a stratified sample using Krippendorff’s alpha [17] as an index of agreement suitable for qualitative content distinctions, with a priori acceptance set at ≥ 0.80 before full-set coding. Discrepancies were resolved by consensus with the codebook refinement. We also triangulated interpretations against contemporaneous policy and program documents to situate the findings. Reporting followed the Standards for Reporting Qualitative Research (SRQR), with Consolidated Criteria for Reporting Qualitative Research (COREQ) elements referenced where applicable to internet-mediated qualitative data [18].

Ethical considerations followed the current guidance for Internet research. We analyzed only publicly available content, did not contact users, and removed or paraphrased potentially identifying details in reported excerpts. All usernames/handles were redacted at the source, and screenshots were not reproduced. Given the health-related and quasi-consultative nature of the discussions, particular care was taken to protect data privacy and confidentiality, recognizing that public availability does not eliminate the expectations of privacy. Informed consent was not sought in line with the guidance for observational research using public online data; ethical justification relied on non-intervention, strict anonymization, and harm-minimization principles. Decisions were guided by the Association of Internet Researchers (AoIR) 3.0 principles and the British Psychological Society’s ethics guidance for Internet-mediated research, emphasizing context, user expectations, harm minimization, and data stewardship [19].

Finally, to respect linguistic equity and reduce interpretive bias, we treated language as an analytic domain and explicitly examined how Sinhala-dominant communication and ad hoc Tamil responses shaped participation and help-seeking. This was methodologically important given the evidence within the thread itself of requests for Tamil/English information and later Tamil expert posts.

## Results

**Fig. 1:**
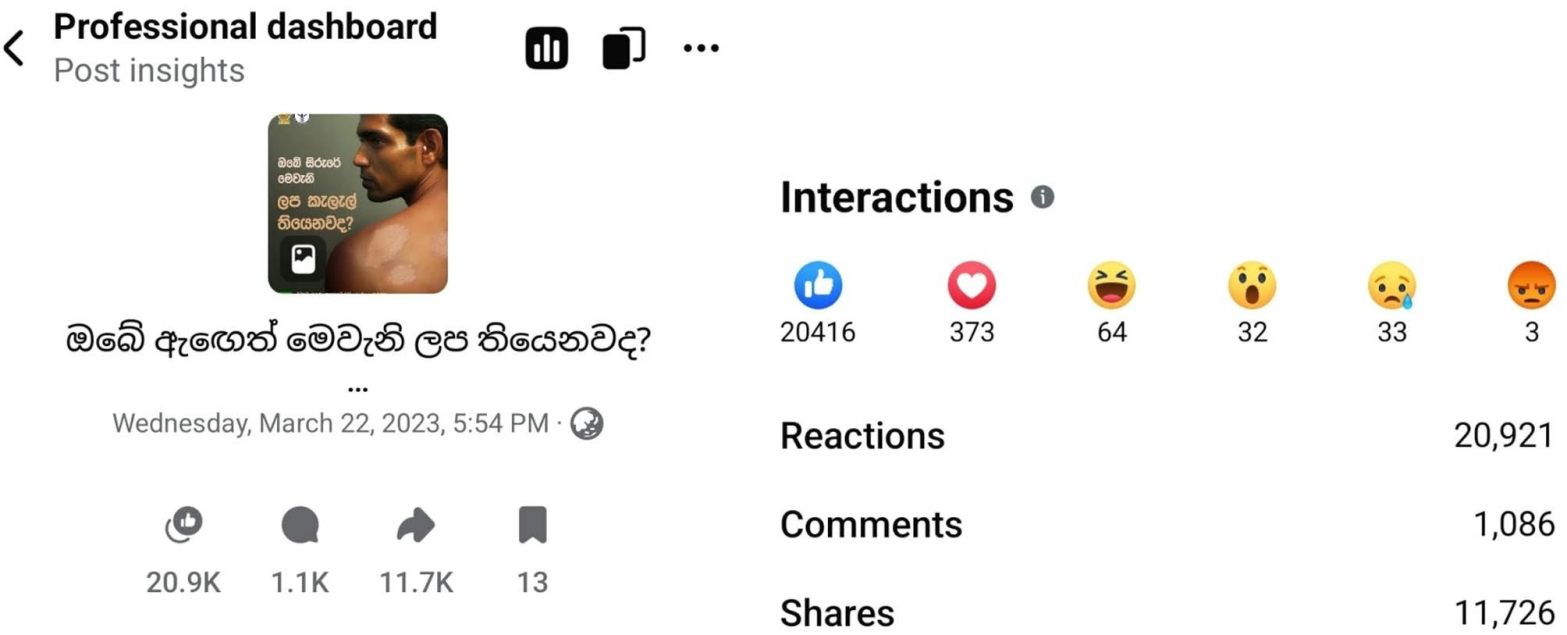
Reach and engagement of the post

In this case, the post generated significant public interaction and showed features consistent with our ‘digital frontline’ description, including real-time expert guidance and clear signposting to the WhatsApp contact route. Figure 1 illustrates the quantitative evaluation of reach and engagement.

The Facebook thread functioned as an organically emergent ‘digital frontline’ in the sense defined above, evolving from a single awareness post into a live public health help desk for the community. Across 806 public comments and replies, identified experts anchored the discourse, and the embedded WhatsApp line received over 150 inbound contacts within the first 24 h, as reported by the Dr. K. D. N. Prasad Ranaweera (Anti-Leprosy Campaign; personal communication, March 2023), and downstream clinical outcomes were not independently verified. These features were associated with a shift from diffuse engagement to referral-oriented interactions.

Participant roles coalesced into four recurrent archetypes: (1) authoritative experts who corrected misinformation and issued clear calls to action; (2) anxious inquirers seeking help for themselves or family members; (3) grateful beneficiaries who returned to validate advice and outcomes; (4) and casual interlocutors whose light banter amplified reach but sometimes trivialized illness. This typology structured the cadence and content of the conversation.

Conversation flows followed a hub-and-spoke pattern: most substantive queries were funneled to expert accounts, while lay replies typically reinforced official guidance, often repeating the WhatsApp referral, thereby appearing to preserve informational integrity and minimize peer-to-peer speculation. This community amplification suggests the progressive internalization of expert messages and contributes to maintaining a credible information environment.

Within this structure, the thread operates in a manner analogous to a real-time triage system. Experts routinely de-escalated non-leprosy concerns, clarified common differentials, and escalated probable or urgent presentations, particularly for children, into clinical care with specific service directions. An exemplar involved a one-year-old with a periocular lesion, where the expert’s response was followed by an immediate referral to a named dermatology clinic.

Thematically, information seeking dominated and exposed recurrent knowledge gaps. Users frequently queried anatomic distribution, lesion color and sensation, and duration from exposure to symptoms; confusion with psoriasis, tinea versicolor (“aluham”), and post-inflammatory marks was common, emphasizing the need for differential cues in public messaging.

The lived experience narratives added depth and urgency. Accounts of repeated misdiagnosis culminating in specialist confirmation of leprosy, as well as descriptions of advanced, disabling disease in family members, framed the human cost of diagnostic delay and aligned with the campaign’s focus on early detection and guided referral. Concurrent posts reflected intense parental and caregiver anxiety, particularly when children were involved in the posts.

Stigma emerged in two intertwined forms. Fear-based stigma echoed historic concerns about contagion and deformity, visible in precautionary questions and a preference for semi-anonymous online advice. More pervasive was “normalized” stigma, joking, and tagging friends as having leprosy, which risked reframing symptoms as punchlines and potentially deterring timely help seeking. Qualitative tracing suggested that this trivialization may suppress both bystander concern and self-referral intentions, a barrier as consequential as overt fear.

The expert communication style, which was consistently empathetic, clear, accountable, and action-oriented, was prominent throughout the interactions. Across multiple micro-episodes, expert interventions were followed by observable shifts in sentiment from anxiety and confusion to reassurance and gratitude, while channeling users toward clinic or WhatsApp pathways. The cumulative pattern suggested the emergence of a community-supported “echo chamber of credibility,” where lay participants began to pre-empt questions by tagging experts and echoing referral instructions, thereby marginalizing sporadic misinformation.

Finally, language accessibility emerged as a critical equity constraint. Although the discourse was predominantly in Sinhala, users explicitly requested Tamil or English explanations, and the clinic’s later Tamil responses appeared reactive rather than planned, which is problematic given the higher burdens in Tamil-speaking regions. This gap emphasizes the need for a multilingual strategy within otherwise effective digital pipelines.

Taken together, the qualitative evidence indicates that expert-moderated social threads can be associated with credible, sentiment-shifting triage-like interactions that align public engagement with pathways toward clinical care while revealing addressable bottlenecks, most notably differential diagnosis confusion, normalized stigma, and language exclusivity, which should be explicitly targeted in subsequent campaigns.

## Discussion

In this single case, a government-led Facebook thread, actively moderated by dermatology experts and coupled with a dedicated WhatsApp line, showed ‘digital frontline’ features and was associated with a shift toward referral-oriented help-seeking. The quantitative metrics of reach and engagement are summarized in Fig. 1, showing that the post reached a substantial audience and generated widespread interaction. In a single post, 806 public comments/replies were accrued, and Anti-Leprosy Campaign program monitoring recorded over 150 inbound WhatsApp contacts within 24 h (Dr K. D. N. Prasad Ranaweera, personal communication, March 2023). This mechanism aligns tightly with the WHO’s emphasis on early diagnosis and prompt multidrug therapy to prevent disability and reduce transmission, and with the Global Leprosy Strategy’s pillars, which include integrated case detection and zero discrimination [13].

Mechanistically, the thread illustrates how expert participation may reconfigure social media from a broadcast channel to an interactive triage system. Contemporary communication science indicates that “engagement” is the key driver of impact in digital public health campaigns, displacing simple exposure or awareness as the primary mechanism of effect [20]. Our case study goes a step further by demonstrating how engagement can be linked to a concrete clinical step (WhatsApp contact), consistent with the pathway from exposure to engagement to behavior change, as described in a recent systematic model of social media effects [20]. Comparable evidence from low- and middle-income settings demonstrates that lightweight messaging tools, such as WhatsApp, are acceptable and feasible conduits for triage and referral, supporting their use as the final mile in digital-to-clinical pipelines [21, 22].

Simultaneously, the discourse surfaces a dual stigma ecology with direct implications for case-finding. Fear-based stigma was visible in anxious, semi-anonymous queries about contagion; more insidious was “normalized” trivialization, with friends tagging each other and joking about leprosy, which may risk reframing early signs as punchlines, potentially suppressing both bystander concern and self-referral [23]. This pattern is consistent with Sri Lanka’s emerging stigma literature: the validation of a Sinhalese SARI scale underscores the salience of perceived stigma for delayed care, and new clinic-based evidence documents substantial stigma burdens among people affected by leprosy [24]. Addressing both overt fear and casual trivialization should, therefore, be an explicit aim of communication design [24]. To overcome stigma-related barriers to engagement, social media awareness campaigns should normalize passive participation, actively moderate stigmatizing or trivializing content, and provide clear pathways to private or semi-anonymous follow-up, thereby reducing fears of identity disclosure while supporting early case finding [22, 23].

Equity considerations were equally important. Although experts responded promptly and empathetically, the thread was largely Sinhala-dominant; Tamil/English explanations appeared late and ad hoc despite Tamil-speaking provinces’ high burden. This linguistic skew risks excluding populations where intensified case-finding is most needed and runs counter to the Strategy’s rights-based commitment to zero discrimination. A proactive multilingual plan for simultaneous content and moderation in Sinhala, Tamil, and English, mirrored in WhatsApp staffing, should be treated as a core design requirement rather than an enhancement [12].

The findings also refine what “good” expert communication looks like in open social spaces is. Named experts anchored the hub-and-spoke flow of substantive questions, delivering concise and accountable answers and clear next steps. Over time, the community appeared to echo and amplify this guidance, creating an “echo chamber of credibility” that contributed to marginalizing sporadic misinformation. This mixture of authority, empathy, and action orientation appears to be central to moving users along the help-seeking pathway while preserving trust [25].

### Strengths and limitations

The strengths of this study include the naturalistic, real-time observation of a population-scale conversation anchored by verified clinicians, the ability to trace micro-episodes from initial anxiety to reassurance and referral within the same public record, and the documentation of reported initial WhatsApp contact within 24 h (Dr K. D. N. Prasad Ranaweera, personal communication, March 2023). These features provide unusually rich visibility into how expert-moderated social media can act as a near-costless triage infrastructure during periods when traditional outreach is constrained.

The limitations of this study warrant caution. First, the analysis is based on a single embedded case study of one Facebook awareness post, which is inherently platform-specific and observational in nature; therefore, observed patterns may not generalize across diseases, populations, or digital networks, nor support causal inferences. Furthermore, the term ‘digital frontline’ is used here as an analytic description drawn from this case, not as a formally evaluated intervention model, and the study cannot establish effectiveness or generalizability. Second, the data were derived exclusively from publicly visible comments, reflecting a self-selected subset of users; this introduced potential selection bias, as silent audiences were not observable, private interactions were inaccessible, and clinical outcomes beyond initial WhatsApp contact could not be independently verified. We did not have access to the content of WhatsApp communications or to subsequent follow-up records; therefore, interactions beyond the initial contact period could not be examined. Third, digital access inequities, including differential access to smartphones, Internet connectivity, and digital literacy, may have further shaped who was able or willing to engage with the content. Fourth, engagement and visibility are shaped by platform algorithms and the ephemeral nature of social media threads, potentially influencing who encountered, interacted with, or acted upon the content and limiting future retrieval or replication. Additionally, because the thread was captured more than two years after the original post, some comments may have been edited or deleted prior to capture, and algorithm-driven visibility may have influenced which interactions remained prominent in the observable thread. Therefore, the analysis reflects the publicly accessible discussion at the time of capture rather than the complete historical record of the conversation. Finally, although ethical safeguards for public-domain analysis were followed, Internet-mediated research continues to raise evolving questions regarding contextual expectations, consent, and potential harm, which should remain central to protocol design and interpretation [25].

The policy implications follow directly. For Sri Lanka’s Anti-Leprosy Campaign and Ministry of Health, piloting similar expert-moderated threads could include pre-assigned moderators, standard triage responses, multilingual coverage, and measurable response-time expectations, alongside a WhatsApp back-end. Any wider adoption should be paired with prospective evaluation linking digital contacts to clinic records with safeguards. Integration with routine surveillance and the National Strategic Plan 2021–2025 would allow digital signals to trigger targeted outreach in high-prevalence districts, explicitly advancing the Global Strategy’s pillars on integrated detection and zero discrimination. In parallel, communication should incorporate stigma-countering creatives that address both fear and trivialization, and deploy patient testimonials to resonate with the need to humanize the cure and reduce delay [12].

Future work should prospectively link digital interactions to clinic records (with safeguards) to estimate the impact on diagnostic intervals and disability at presentation, compare multilingual versus monolingual deployments, and test whether expert-moderated models outperform unmoderated campaigns in reducing misinformation and accelerating appropriate referral. Given the low marginal cost and high acceptability of messaging-based triage in other LMIC pathways, scaling this approach offers a pragmatic route to closing the gap between awareness and care while Sri Lanka pursues “Towards Zero Leprosy” [13].

## Conclusions

This case study suggests that a government-led, expert-moderated Facebook thread connected to a specialized WhatsApp service may resemble a ‘digital frontline’ as defined in this paper, supporting rapid public guidance and signposting to care. The initiative not only elicited rapid public interaction but also aligned with engagement in tangible help-seeking actions. Expert involvement grounded the discussion, transformed apprehension into confidence, disputed false information, and promoted the community’s enhancement of reliable advice. The investigation identified enduring obstacles, such as fear-induced and normalized stigma, deficiencies in knowledge concerning differential diagnosis, and linguistic disparities, underscoring the necessity for multilingual, stigma-aware communication tactics.

This case study indicates that expert-moderated digital threads can enhance conventional communication, functioning as an affordable, scalable triage system during times of limited mobility or restricted access to health services. Establishing the digital frontline approach with systematic moderation, multilingual assistance, and quantifiable performance indicators could enhance national leprosy control initiatives and expedite advancements toward Sri Lanka’s “Towards Zero Leprosy” objectives. Subsequent research connecting digital engagement to clinical results will enhance the validation of this methodology and guide its replication in various low- and middle-income settings.

### Recommendations

This case study outlines realistic measures to enhance digital outreach for leprosy in Sri Lanka. If the Ministry of Health chooses to pilot similar arrangements, key elements to test include preassigned expert moderators, standard triage responses, multilingual coverage, and clear response targets, alongside a WhatsApp contact route and an evaluation plan that links digital contacts to clinic records with safeguards. Communication efforts must tackle both fear-induced and normalized stigma, include patient testimonials, and provide multilingual accessibility in Sinhala, Tamil, and English languages. Encouragement of community reinforcement of professional guidance should occur alongside vigilance against disinformation. Ultimately, a prospective study connecting digital engagement with clinical outcomes is crucial to evaluate its impact on diagnostic intervals, presentation disability, and effectiveness compared to unmoderated campaigns.

### Disclosure of AI usage

During the preparation of this manuscript, the authors used AI tools, including ChatGPT 5-Thinking, to assist with writing, summarizing, polishing the language, condensing the text, and improving clarity. All outputs were reviewed, adapted, and integrated into the final work by the authors, who take full responsibility for all content presented herein.

## Data Availability

The datasets used in the current study are available from the corresponding author upon reasonable request.

## Abbreviations

AoIR: Association of Internet Researchers
ALC: Anti Leprosy Campaign
COREQ: Consolidated Criteria for Reporting Qualitative Research
LMIC: Low-and middle-income countries
MDT: Multidrug therapy
NHSL: National Hospital Sri Lanka
NTD: Neglected tropical disease
SRQR: Standards for Reporting Qualitative Research
WHO: World Health Organization
